# 高效液相色谱-气相色谱在线联用同时测定土壤中饱和烃和芳香烃

**DOI:** 10.3724/SP.J.1123.2021.02011

**Published:** 2021-08-08

**Authors:** Lingling LIU, Bingning LI, Yanwen WU

**Affiliations:** 北京市理化分析测试中心, 北京市食品安全测试工程技术研究中心, 北京 100094; Beijing Center for Physical & Chemical Analysis, Beijing Food Safety Analysis and Testing Engineering Research Center, Beijing 100094, China; 北京市理化分析测试中心, 北京市食品安全测试工程技术研究中心, 北京 100094; Beijing Center for Physical & Chemical Analysis, Beijing Food Safety Analysis and Testing Engineering Research Center, Beijing 100094, China; 北京市理化分析测试中心, 北京市食品安全测试工程技术研究中心, 北京 100094; Beijing Center for Physical & Chemical Analysis, Beijing Food Safety Analysis and Testing Engineering Research Center, Beijing 100094, China

**Keywords:** 高效液相色谱-气相色谱在线联用, 饱和烃, 芳香烃, 土壤, on-line high performance liquid chromatography-gas chromatography (HPLC-GC), saturated hydrocarbons, aromatic hydrocarbons, soil

## Abstract

为加强对土壤中石油烃类污染物的风险管控,生态环境部已将石油烃类列为土壤中的重点监测项目。石油烃源于石油与合成油,是涵盖一定碳数范围的碳氢化合物,主要分为饱和烃和芳香烃两大类。芳香烃通常是高度烷基化的单环、双环与多环芳烃,其对人和动物的毒性较饱和烃大很多,因此,仅仅测定土壤中总石油烃含量难以准确评估其环境毒性。目前环境领域的标准方法尚未区分土壤中饱和烃和芳香烃。该研究针对土壤样品的基质干扰特点,对样品的提取和净化环节进行了优化,并且应用高效液相色谱-气相色谱在线联用(HPLC-GC)技术,建立了同时测定土壤中饱和烃和芳香烃的方法。其中,提取方法选择正己烷-乙醇(1:1, v/v)以固液比1:4常温振荡提取1 h,然后水洗去除乙醇,取正己烷层提取液净化;净化方法选择自制硅胶柱,以正己烷-二氯甲烷(8:2, v/v)洗脱;洗脱液经浓缩注入HPLC-GC分析,以内标法同时测定试液中的饱和烃和芳香烃,方法的定量限为0.4 mg/kg。该方法经过土壤石油烃标准物(SQC-116)验证,测定值在证书提供的可信区间内,相对误差(RE)为10.6%,相对标准偏差(RSD)为1.4%,说明方法准确可靠且精密度达到分析要求。最后,该文采用建立的方法检测了北京地区的5个土壤样品,结果表明:5个样品均含有饱和烃(C_10_~C_40_),其含量范围为3.3~32.1 mg/kg;其中4个样品中检出芳香烃(C_10_~C_40_),其含量范围为0.8~4.3 mg/kg;此外,通过谱图分析还可以初步判别烃类物质的污染来源。

## Abstract (en)

To strengthen regulation for mitigating the risk posed by petroleum contaminants in soil, the Ministry of Ecology and Environment of the People’s Republic of China has classified petroleum hydrocarbons as a key monitoring item for regulatory contamination monitoring. Petroleum is principally derived from petroleum and synthetic fuels, which contain an extremely high content of hydrocarbon compounds that have varied boiling points. These compounds are chemically classified primarily as saturated and aromatic. Aromatic hydrocarbons are typically highly alkylated monocyclic, bicyclic, and polycyclic, which are more toxic to human and animal life than saturated hydrocarbons. Because of the significant toxicological differences among the various hydrocarbons, it is difficult to accurately assess their environmental toxicity by only determining the total content of petroleum in soil. However, there are no analytical methods for the simultaneous determination of saturated hydrocarbons and aromatic hydrocarbons in soil according to Chinese standards.

In this study, extraction and purification procedures were completely optimized depending on the matrix of the soil samples. The advanced analytical technique of on-line high performance liquid chromatography-gas chromatography (HPLC-GC) was performed after sample preparation for the simultaneous determination of saturated hydrocarbons and aromatic hydrocarbons in soil. For the extraction, *n*-hexane/ethanol (1:1, v/v) was chosen as the extraction solvent. The ratio of solid sample (soil) to the solvent was chosen as 1:4, and extraction was performed once at room temperature, for 1 h. Water was then added and mixed to remove ethanol from the extracts, and the upper *n*-hexane layer was separated; thus, the petroleum hydrocarbons in the samples were completely extracted. However, the oils and fats in the matrix of the soil sample were also simultaneously extracted. Because of the limited retention capacity of the HPLC column, the presence of oils, fats, and other interferents would affect the subsequent determination of saturated hydrocarbons and aromatic hydrocarbons. Therefore, an additional purification step is required before sample injection into the HPLC-GC equipment. In this study, purification was performed using a lab-made silica gel column, which is commonly used for the determination of saturated hydrocarbons and aromatic hydrocarbons in food. The purification column was conditioned and eluted with an 8:2 ratio (v/v) of *n*-hexane to dichloromethane after sample loading. Subsequently, the eluent was concentrated and injected into the HPLC-GC equipment for analysis. The flame ionization detector (FID) is ideal for petroleum hydrocarbons quantification because of its nearly identical responses to all hydrocarbons; hence, with the FID, reference standards are not required for quantification, and internal standards are typically used for quantifying the total hydrocarbon content. In this study, cyclohexylcyclohexane (Cycy) and 2-methylnaphthalene (2-MN) were used as internal standards for determining the saturated and aromatic hydrocarbons, respectively. The limit of quantification (LOQ) of this proposed method was 0.4 mg/kg. Moreover, the suitability of the method was verified by comparing the obtained content against the soil petroleum hydrocarbon standard (SQC-116); the measured value was found to be within the credible interval provided by the standard. The relative error (RE) was 10.6% with a relative standard deviation (RSD) of 1.4%, which indicates that the proposed method is accurate and reliable, and the precision meets analytical requirements. Finally, the method was applied to the determination of hydrocarbons in five soil samples from the Beijing area. Saturated hydrocarbons (C_10_-C_40_) were detected in all five samples, with contents ranging from 3.3 to 32.1 mg/kg, while aromatic hydrocarbons (C_10_-C_40_) were detected in four samples, with contents ranging from 0.8 to 4.3 mg/kg.

HPLC-GC combines the high selectivity of HPLC with the high separation efficiency of GC, and as demonstrated in this study, can be used for the simultaneous determination of saturated and aromatic hydrocarbons in soil. The source of hydrocarbon contamination can also be preliminarily identified by chromatographic analysis.

石油作为能源和化学工业的原材料在全球的应用十分普遍,然而,其开采、运输、储存等过程均可能对环境造成污染风险,此外,一些人为因素导致的环境污染也不容忽视,如油罐泄露、油轮事故、废水排放等^[1]^。烃类污染物不易降解,会持续影响生态系统和人类健康。因此,土壤中的石油污染一直是环境监测的重点^[2,3]^。

石油的化学组成非常复杂,包含不同结构类型的碳氢化合物,即烃类化合物。石油烃的结构类型包括直链或支链脂肪族、脂环族和高度烷基化的芳香族,其中,直链或支链脂肪族、脂环族统称为饱和烃(saturated hydrocarbons, SH),含有烷基化芳香族则统称为芳香烃(aromatic hydrocarbons, AH)^[4,5]^。由于石油烃的碳数从十几到几十,因此其包含的化合物数量巨大,无法将其分离成单个物质分析,只能进行总量测定。红外光谱法、紫外和荧光分光光度法等分子光谱分析方法均曾经用于石油烃的总量测定^[6,7,8,9]^,但均存在局限性。紫外和荧光方法只能测定芳香烃,红外光谱法虽然可以同时测定饱和烃和芳香烃,但需要使用剧毒的四氯化碳或四氯乙烯。更为关键的是,由于石油烃的污染来源复杂且多样,不同样品中石油烃的化学组成迥异,无法找到合适的标准品与之匹配。分子光谱法缺乏与目标物匹配的标准品,其测定结果无法真实反映实际情况。氢火焰离子化检测器(FID)对烃类物质的响应几乎完全一致,因而其定量结果与标准品无关,只需任一烃类化合物作为内标即可对不同来源的石油烃污染物进行准确定量^[10]^。因此,近年来气相色谱法(GC)成为环境中石油烃分析的新标准^[11,12,13,14,15,16]^。

然而,GC-FID缺乏选择性,无法区分饱和烃与芳香烃,石油烃中不同组分的毒性差别很大,如饱和烃有蓄积作用,导致微型肉芽肿的形成,而芳香烃可能致畸和致癌风险^[17]^。为了分别测定饱和烃和芳香烃,美国马萨诸塞州的标准方法是将样品通过二氯甲烷萃取,经硅胶柱分离得到饱和烃和芳香烃^[5]^。不过,该方法消耗溶剂多、步骤繁琐、操作难度较大。目前,食品领域采用在线联用高效液相色谱-气相色谱(on-line high performance liquid chromatography-gas chromatography, HPLC-GC)技术测定其中的矿物油污染物,该技术有效整合了烃类分析中的净化、分离与检测步骤,其中HPLC的硅胶柱可以吸附油脂等极性物质,同时分离饱和烃和芳香烃,然后通过阀切换、预柱与溶剂排空阀组成的HPLC-GC接口,将分离出来的饱和烃和芳香烃全部送入GC分析,真正实现了全样品分析,从而极大地提升了分析灵敏度;此外,这套系统通常配置两套完全相同的通道(每个通道均由一套预柱、三通、溶剂放空阀、分析柱和FID组成),一次进样即可同时实现饱和烃和芳香烃的测定,成倍地提高了分析效率。HPLC-GC的应用有效减少了样品量和试剂消耗,简化了实验步骤,避免了污染引入,从而提高了方法的准确性和结果的重现性^[10,18]^。我们前期将该技术成功运用于奶粉、大米、巧克力等食品中矿物油的测定^[19,20,21]^,结果发现,不同样品的基质干扰不同,其涉及的提取与净化方法各有差异。以提取方法为例,大部分干物质可以直接用正己烷浸泡提取,湿物质则需要事先去除水的干扰,而一些喷雾干燥的样品则需要水解处理才能提取完全^[22]^,等等。本研究将HPLC-GC用于土壤中饱和烃与芳香烃的测定,需要根据土壤基质的干扰情况对样品前处理方法进行优化。首先本文对提取方法进行了考察和优化,包括提取溶剂、时间、温度和次数等提取条件;其次,由于HPLC-GC中HPLC硅胶柱的吸附容量有限,本文增加离线的固相萃取(SPE)净化步骤,考察优化了去除提取液中油脂等极性干扰物的净化条件;最后,采用HPLC-GC技术建立了同时测定土壤中饱和烃和芳香烃的方法。此外,本文还通过谱图分析探索了石油烃的污染来源,这些均为了解环境中石油烃的污染情况与制定治理策略提供数据支撑。

## 1 实验部分

### 1.1 仪器、试剂与材料

HPLC-GC联用仪器:包括配备二元泵和UV检测器的LC 20A液相色谱仪,带有FID的GC 2010 plus气相色谱仪(日本Shimadzu公司), HPLC-GC接口(德国Axel Semrau公司)和PAL自动进样器(瑞士CTC公司)。

正己烷、二氯甲烷、无水乙醇、甲苯均为色谱纯(美国Fisher Scientific公司);无水硫酸钠为分析纯(国药集团化学试剂有限公司);硅胶(0.063~0.200 mm),使用前400 ℃下活化16 h(德国Merck公司)。

SQC-116的土壤石油烃标准物(标准值2541 mg/kg,可信区间938~3750 mg/kg)购自美国NSI Lab solutions公司;9种饱和烃/芳香烃混合标准溶液(15~60 mg/L,溶剂是正己烷和/或甲苯,分别作为内标使用):正十三烷(*n*-C_13_)的质量浓度为15 mg/L,正十一烷(*n*-C_11_)、环己基环己烷(Cycy)、戊基苯(5B)、1-甲基萘(1-MN)、2-甲基萘(2-MN)、1,3,5-三叔丁基苯(TBB)的质量浓度均为30 mg/L, 5*α*-胆甾烷(Cho)和苝(Per)的质量浓度均为60 mg/L,以上9种内标物质以及C_7_~C_40_正构烷烃混合标准品(1000 mg/L)均购自美国Sigma-Aldrich公司。其中*n*-C_11_和5B分别用于饱和烃和芳香烃部分的挥发损失考察;Cycy和2-MN分别为饱和烃和芳香烃的定量内标;*n*-C_13_和1-MN用于考察内标物Cycy和2-MN的响应信号的准确性;此外,Cho、TBB和Per用于监控HPLC分离情况,它们分别为饱和烃流分的末端、芳香烃的开端与末端标记物。

### 1.2 试样制备

1.2.1 试样提取

称取5.0 g土壤样品,加入20 mL正己烷-乙醇(1∶1, v/v)混合试剂和30 μL饱和烃/芳香烃混合标准溶液,振荡1 h,然后加入20 mL去离子水,水洗去乙醇,取正己烷相。

1.2.2 试样净化

将2 g活化硅胶装入玻璃层析柱,硅胶顶部覆盖1 g无水硫酸钠,以10 mL正己烷-二氯甲烷混合溶剂(8∶2, v/v)平衡柱床,然后加入上述提取液,待液面近干,以10 mL左右的正己烷-二氯甲烷混合溶剂(8∶2, v/v)淋洗,收集洗脱液并浓缩至约1 mL,注入HPLC-GC分析。

### 1.3 HPLC-GC分析

1.3.1 HPLC条件

Allure Si色谱柱(250 mm×2.1 mm, 5 μm, 6 nm,美国Restek公司);流动相A为正己烷,B为二氯甲烷;梯度洗脱:0~0.1 min, 100%A(流速为0.3 mL/min); 0.1~6.2 min, 70%A(流速为0.3 mL/min); 6.2~15.2 min, 100%B(反冲,流速为0.5 mL/min); 15.2~25.2 min, 100%A(流速为0.5 mL/min); 25.2~30 min, 100%A(流速为0.3 mL/min)。HPLC运行过程的流动相变换与饱和烃/芳香烃流出通过紫外检测器(230 nm)监测,进样量为50 μL。

1.3.2 HPLC-GC接口

基于HPLC-GC联用技术^[21]^,经HPLC分离后分别得到450 μL的饱和烃(2.0~3.5 min)和芳香烃(4.5~6.0 min)。两段流分通过阀切换,以氢气为载气被导入GC分析。GC仪器配备了由预柱(Restek MXT无涂层毛细管预柱,10 m×0.53 mm)和分析柱(Restek MXT毛细管柱,15 m×0.25 mm×0.25 μm)组成的两个平行通道。饱和烃和芳香烃各自进入一个通道,预柱与分析柱之间通过三通与溶剂排空阀连接。溶剂排空阀在LC流分阀切换前0.5 min开启,转移结束后0.3 min关闭。转移到GC系统的饱和烃和芳香烃中大部分溶剂通过溶剂排空阀去除,剩余少量溶剂与浓缩的溶质聚集在分析柱入口,进行后续GC分离和测定。

1.3.3 GC条件

程序升温的初始温度60 ℃(保持6 min),以15 ℃/min升温至120 ℃,再以25 ℃/min升温至370 ℃(保持6 min)。FID温度为380 ℃;辅助气、燃烧气和助燃气分别为氮气、氢气和空气,流速分别为30、40和400 mL/min。

### 1.4 数据分析

饱和烃和芳香烃在GC-FID谱图中均呈一定沸程范围的驼峰,定量计算时,饱和烃/芳香烃的含量通过计算谱图基线与驼峰之间的面积得到,基线取决于样品空白的GC谱图;驼峰上方的内标物尖峰定量计算时扣除^[10]^。采用内标法对饱和烃/芳香烃进行定量,饱和烃的定量内标物为环己基环己烷(Cycy),芳香烃的定量内标物为2-MN。

### 1.5 空白实验

除不加土壤样品外,按照1.2节和1.3节进行操作,得到样品空白实验的HPLC-GC谱图,该谱图不含干扰石油烃测定的驼峰,基线近似平直,基线偏移的高度与石油烃信号的高度之比不超过四分之一。

## 2 结果与讨论

### 2.1 提取

2.1.1 提取溶剂的选择

根据相似相溶原理,正己烷、正庚烷、二氯甲烷等弱极性溶剂可用于提取石油烃。由于土壤中含有不同程度的水分,土壤颗粒孔隙中的水会阻止石油烃扩散至提取溶剂,因而需要对湿样品进行事先脱水。然而,通常采用的蒸发脱水容易导致挥发性烃类物质的损失。因此,通过加入与水互溶的极性溶剂,利用其渗透固体颗粒表面的水层来促进湿样品中石油烃的溶出。国际标准ISO 16703采用丙酮-正庚烷(2∶1, v/v)或非极性溶剂(如石油醚、环己烷、正己烷等)提取土壤中的石油烃^[4]^;美国标准EPA 3540C规定土壤/沉积物的提取溶剂采用正己烷-丙酮(1∶1, v/v)或二氯甲烷-丙酮(1∶1, v/v)^[23]^;我国环境行业标准HJ 1021-2019《土壤沉积物石油烃(C_10_~C_40_)的测定 气相色谱法》选用正己烷-丙酮(1∶1, v/v)或正己烷作为提取溶剂^[16]^。

由于正庚烷的沸点较高,为98.5 ℃,去除溶剂时需要的时间较长;丙酮属于易制毒品,购买受到限制;二氯甲烷则被列入《优先控制化学品名录》,应最大限度减少生产和使用。因此,选取正己烷替代正庚烷和二氯甲烷,以乙醇替代丙酮作为石油烃提取溶剂。本文分别考察和对比了正己烷和正己烷-乙醇(1∶1, v/v)的提取效果。结果表明:正己烷-乙醇(1∶1, v/v)的提取效率(13.9 mg/kg)明显优于正己烷的(5.8 mg/kg)(色谱图见图1);同时乙醇的毒性很低,故本研究选取正己烷-乙醇(1∶1, v/v)作为土壤中石油烃的提取溶剂。

**图1:**
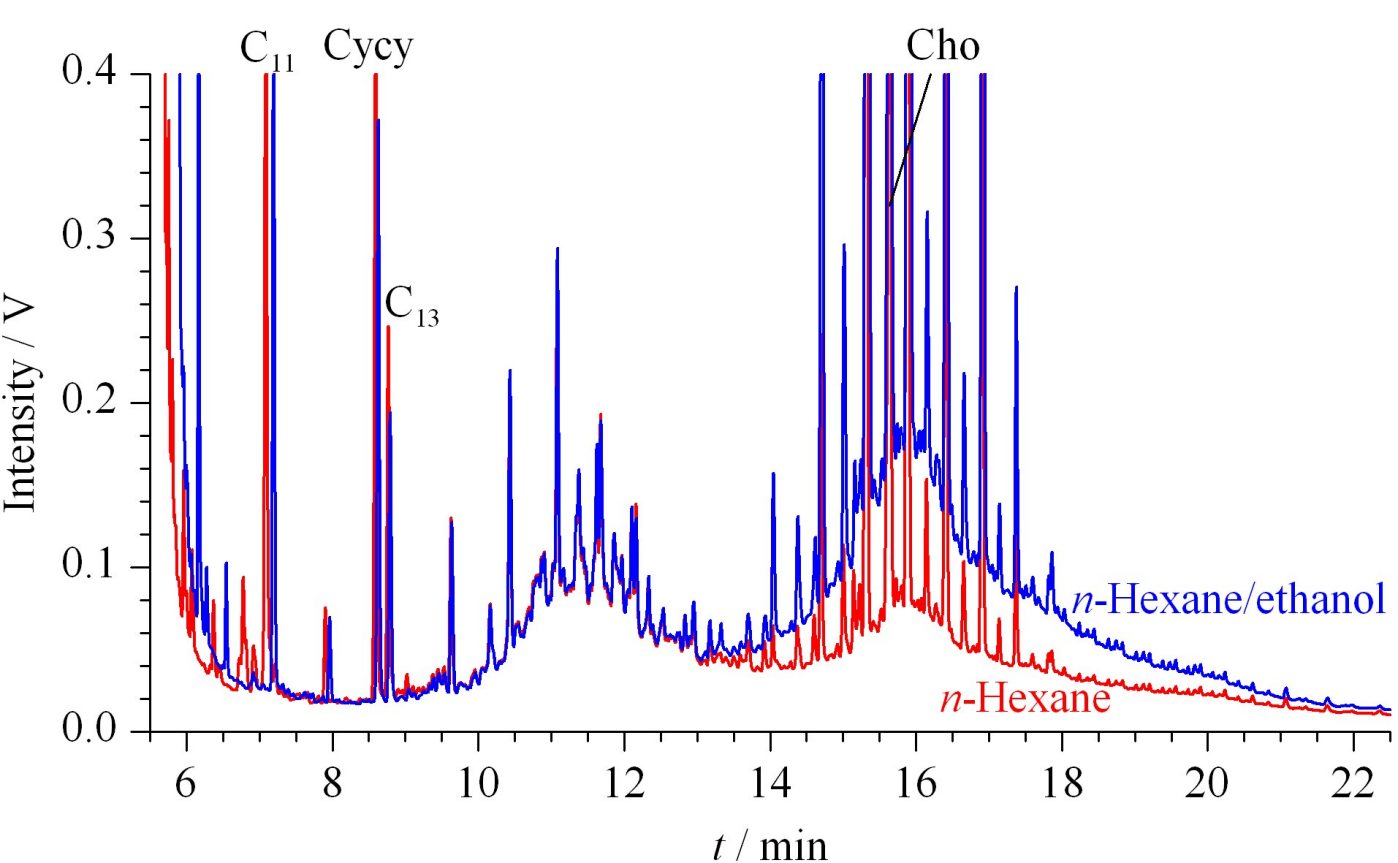
不同溶剂提取同一土壤样品中饱和烃的HPLC-GC谱图

2.1.2 提取级数的优化

为了考察提取是否完全,将土壤样品按照2.1.1节的方法再重复提取一次,然后合并两次提取液。结果发现:提取两次比提取一次的时间和溶剂消耗量均增加一倍,但饱和烃的含量仅增加了4.3%(见表1),说明经过一次提取即可将绝大部分的石油烃提取出来。因此,确定提取级数为一次。

**表1:** 提取级数对同一土壤样品中饱和烃(C_10_~C_40_)测定的影响

Extraction time	Contents/(mg/kg)				RSD/%
	1	2	3	Average	
1	14.0	13.8	13.8	13.9	0.8
2	14.3	15.1	14.2	14.5	3.4

### 2.2 净化

2.2.1 净化填料的选择

提取石油烃时,土壤中的腐殖酸、脂类、色素等极性干扰物也被一同提取出来^[24]^,这些干扰物影响后续的分析,因此,需要对提取液进行净化。硅胶和硅酸镁是常用的净化填料^[25]^,都能够吸附动、植物油等极性干扰物质。本研究采用食品中矿物油分析常用的活化硅胶作为净化填料^[26,27,28,29,30]^,并且考察和优化了硅胶柱的净化条件。

2.2.2 洗脱溶剂的选择

当石油烃提取液进入硅胶柱后,首先流出的是极性最低的饱和烃,随后是极性稍高的芳香烃,最后是甘油三酯和环氧烯烃等极性更高一些的物质。具体细分到饱和烃部分,由于体积排阻效应,首先流出的是大相对分子质量的链烷烃、其次是低相对分子质量的链烷烃和环烷烃;对于芳香烃部分,则首先流出的是高度烷基取代的单环芳烃,其次是高度烷基取代的多环芳烃、最后是低烷基取代或无烷基取代的芳香烃。为了严格控制石油烃的净化以及饱和烃和芳香烃的分离情况,通常以多个化合物标记组分的流出情况,即Cho标记饱和烃的末端,TBB和Per分别标记芳香烃的开端与末端。此外,标准溶液中通常还添加低沸点的*n*-C_11_和5B以分别考察饱和烃和芳香烃部分在整个前处理过程是否存在挥发损失;饱和烃部分的定量内标是Cycy(通常*n*-C_13_的浓度为Cycy的一半,用于考察Cycy的回收率),芳香烃部分的定量内标是2-MN或1-MN(两种化合物的浓度相同)^[18,31]^。

由于HPLC硅胶色谱柱(250 mm×2.1 mm)只能吸附20 mg油脂^[18]^,为了确保HPLC柱的分离性能,提取液在注入HPLC分离饱和烃和芳香烃之前需要预先采用SPE柱净化。将1 mL饱和烃/芳香烃混合标准溶液转移至SPE柱,待上样液近干时,分别用15 mL正己烷和正己烷-二氯甲烷(8∶2, v/v)洗脱,每1 mL洗脱液收集1管,注入GC分析,考察洗脱溶剂能否将饱和烃和芳香烃完全洗脱。研究表明,仅用正己烷洗脱,直至最后1 mL芳香烃末端的标记物Per仍未流出;而采用正己烷-二氯甲烷(8∶2, v/v)洗脱,Per在第5管流出,11管结束(见图2), 99%以上Per集中在5管到10管之间。为了充分回收芳香烃,本研究采用10 mL正己烷-二氯甲烷(8∶2, v/v)混合溶剂作为洗脱液。

**图2:**
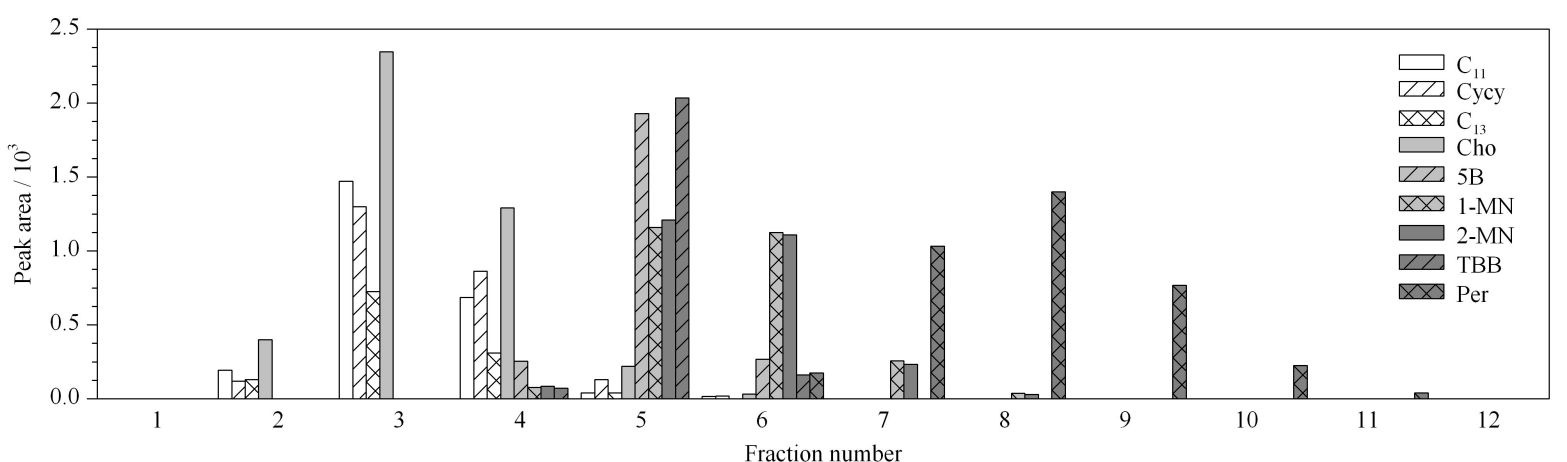
饱和烃和芳香烃(AH)混合标准溶液通过硅胶柱净化后以正己烷-二氯甲烷(8∶2, v/v)混合溶剂洗脱的结果

2.2.3 HPLC-GC分析

将饱和烃/芳香烃混合标准溶液按照上述步骤净化,所得洗脱液浓缩至约1 mL,注入HPLC-GC以1.3节条件分析。结果表明,*n*-C_11_、*n*-C_13_、Cycy和Cho只出现在饱和烃通道中,5B、1-MN、2-MN、TBB和Per只出现在芳香烃通道中;且*n*-C_11_和5B的回收率不低于90%, Cycy和*n*-C_13_的峰面积比例为2∶1, 1-MN和2-MN的峰面积比例为1∶1(见图3)。说明SPE柱净化性能和HPLC柱分离性能满足同时测定土壤中饱和烃和芳香烃的需求。

**图3:**
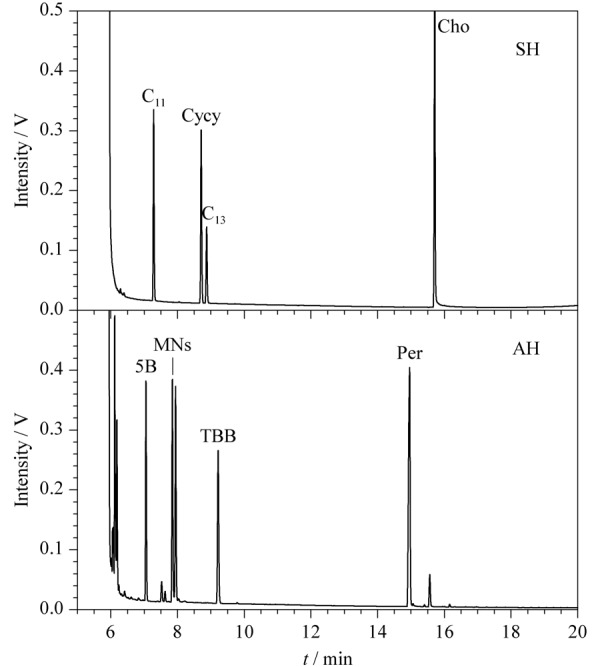
SH/AH混合标准溶液经硅胶柱净化后的HPLC-GC谱图

### 2.3 方法学考察

2.3.1 仪器性能考察

以1.3节条件分析*n*-C_7_~*n*-C_40_正构烷烃混合溶液(见图4), *n*-C_10_、*n*-C_11_…、*n*-C_39_、*n*-C_40_相对于*n*-C_20_的响应因子在0.92~1.05之间,说明分析过程中低沸点目标物没有挥发损失,对高沸点目标物没有歧视,满足分析要求(*n*-C_10_、*n*-C_11_、…、*n*-C_39_、*n*-C_40_相对于*n*-C_20_响应因子应高于0.80)^[4]^。

**图4:**
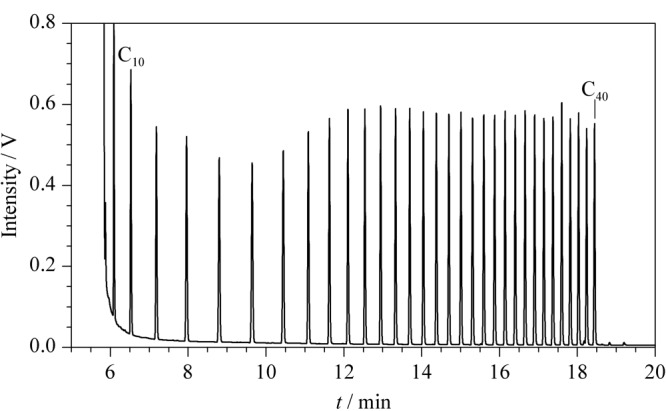
*n*-C_10_~*n*-C_40_正构烷烃混合溶液的HPLC-GC谱图

2.3.2 线性范围考察

分别配制2.5、4、8、20、50、150、250、500 mg/L润滑油(饱和烃与芳香烃的质量比为85.5∶14.5)的正己烷溶液,取50 μL注入HPLC-GC测定。以润滑油系列溶液浓度为横坐标,分别以饱和烃和芳香烃驼峰的峰面积为纵坐标,绘制标准曲线。结果表明:饱和烃在2.1~427.5 mg/L、芳香烃在2.9~72.5 mg/L范围内线性关系良好,线性方程分别为*y*=1045.9*x*-1532.3 (*R*^2^=0.9997)和*y*=146.4*x*-772.3 (*R*^2^=0.9993)。

2.3.3 定量限

由于石油烃的GC-FID谱图呈驼峰,根据仪器噪声计算检出限的方法GB5009.1-2003不适用于本方法。本方法定量限采用文献方法计算,即进入FID的石油烃总量至少达到100 ng才能准确测定^[18]^。本方法称取5 g土壤,经提取、SPE柱净化、浓缩等操作后得到体积为1 mL的供试液,最后取50 μL注入HPLC-GC定量分析,即相当于有0.25 g样品中的石油烃进入FID检测,因而对应的LOQ值为0.4 mg/kg。

2.3.4 准确度和精密度验证

称取0.5 g SQC-116土壤石油烃标准物质7份,用上述方法测定,考察方法的准确度和精密度。测定平均值为2809 mg/kg,与标准值相对误差(RE)为10.6%,在证书提供的可信区间内;相对标准偏差(RSD)为1.4%,表明此方法分析测定土壤中的石油烃具有良好的精密度和准确度。

### 2.4 实际样品分析

本实验采用上述方法,检测了北京地区的5个土壤样品中饱和烃和芳香烃含量,结果见表2。结果表明,5个土壤样品中均检出饱和烃(C_10_~C_40_),其含量范围为3.3~32.1 mg/kg;其中4个样品中检出芳香烃(C_10_~C_40_),其含量范围为0.8~4.3 mg/kg,芳香烃的比例范围为6.3%~13.2%。

**表2:** 土壤样品中饱和烃和芳香烃(C_10_~C_40_)的含量

Sample No.	SH/(mg/kg)	AH/(mg/kg)	Aromatics ratio/%
1	6.6	1.0	13.2
2	32.1	4.3	11.8
3	3.3	<0.4	-
4	13.8	1.1	7.4
5	11.8	0.8	6.3

我们分析了两个土壤样品的谱图(见图5)。结果显示:不同样品的饱和烃与芳香烃呈现出不同的碳数范围分布,其中No.1样品中的饱和烃由两个驼峰组成,低碳数范围以*n*-C_18_为中心,含量为3.0 mg/kg,高碳数范围以*n*-C_29_为中心,含量为3.6 mg/kg(图5a-SH);此外,No.1样品的芳香烃谱图也显示出与低碳数饱和烃(以*n*-C_18_为中心)对应的驼峰,含量为0.6 mg/kg(图5a-AH)。同样,No.5土壤样品也显示出同样碳数范围的饱和烃和芳香烃驼峰(图5b),只是含量不同。通常认为低碳数范围的烃类物质主要来自柴油,而高碳数的烃类物质驼峰是润滑油的典型特征^[32]^。图5的结果表明:土壤中的烃类物质来源复杂,包含不同的碳数范围,其中低碳数范围含有芳香烃污染物。

**图5:**
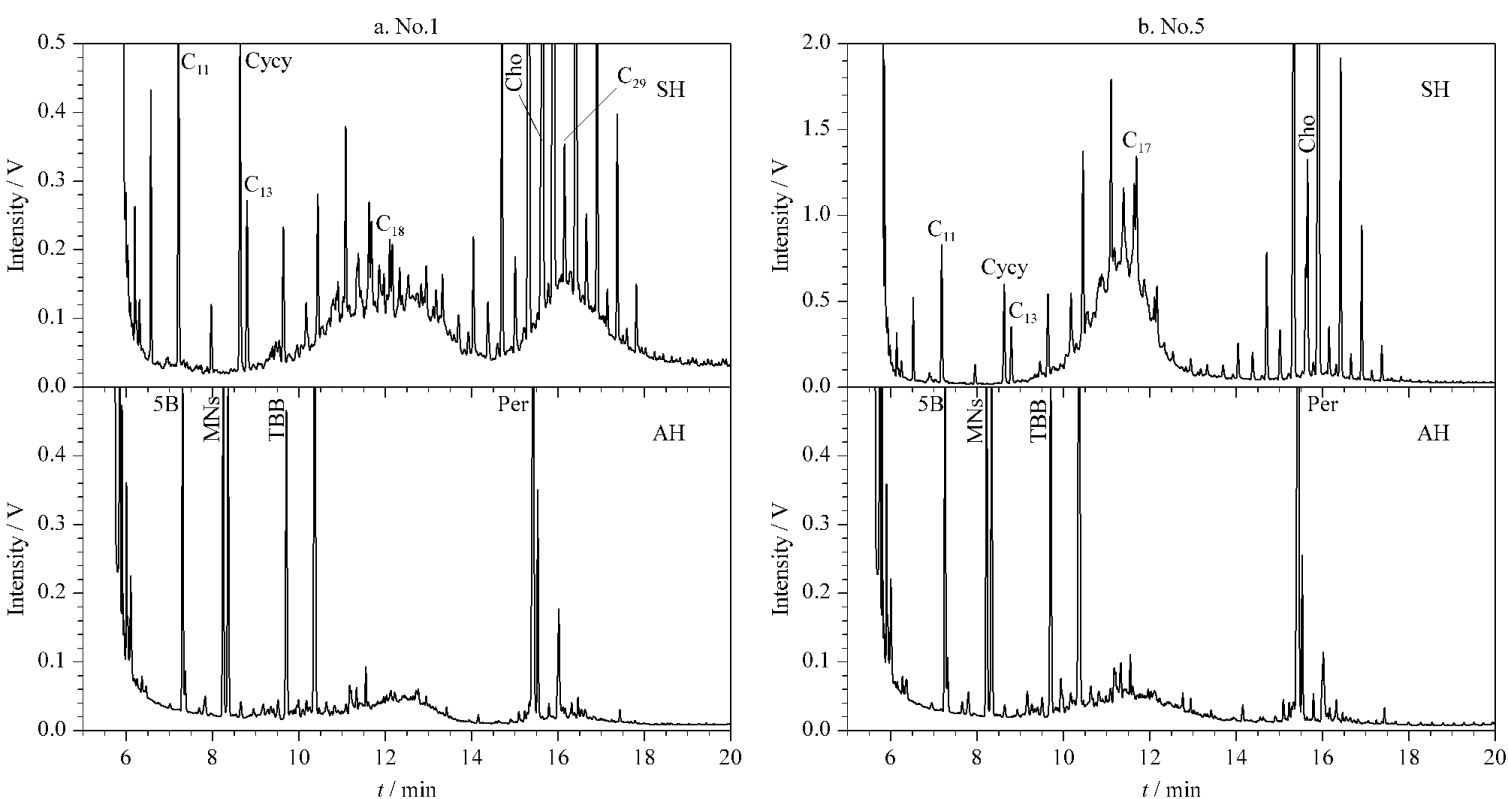
两个土壤样品中SH和AH的HPLC-GC谱图

## 3 结论

本研究建立了高效液相色谱-气相色谱联用法同时测定土壤中饱和烃和芳香烃的方法,针对土壤样品特性,优化了提取和净化两个试样制备环节。该方法整合了石油烃分析的净化、分离与检测步骤,灵敏度高、溶剂消耗少、准确度及精密度良好,适合于土壤中可萃取性石油烃的测定。同时,通过HPLC柱有效分离饱和烃和芳香烃,实现了毒性相对较高的芳香烃的测定,能更好地满足石油烃污染物的监测与评估需要。此外,通过谱图分析还可以探索石油烃的污染来源,有助于进一步提出相应的预防处理措施。
